# The effectiveness of trauma care systems at different stages of development in reducing mortality: a systematic review and meta-analysis

**DOI:** 10.1186/s13017-021-00381-0

**Published:** 2021-07-13

**Authors:** Rayan Jafnan Alharbi, Sumina Shrestha, Virginia Lewis, Charne Miller

**Affiliations:** 1 School of Nursing & Midwifery, La Trobe University, 1st floor, HSB 1, La Trobe University, Bundoora, VIC 3086 Australia; 2 Department of Emergency Medical Service, Jazan University, Jazan, Saudi Arabia; 3 Australian Institute for Primary Care and Ageing, School of Nursing & Midwifery, La Trobe University, Bundoora, VIC Australia; 4 Community Development and Environment Conservation Forum, Chautara, Nepal

**Keywords:** Trauma system, Trauma centre, Traumatic injury, System effectiveness, Mortality, Systematic review

## Abstract

**Background:**

Traumatic injury remains the leading cause of death, with more than five million deaths every year. Little is known about the comparative effectiveness in reducing mortality of trauma care systems at different stages of development. The objective of this study was to review the literature and examine differences in mortality associated with different stages of trauma system development.

**Method:**

A systematic review of peer-reviewed population-based studies retrieved from MEDLINE, EMBASE, and CINAHL. Additional studies were identified from references of articles, through database searching, and author lists. Articles written in English and published between 2000 and 2020 were included. Selection of studies, data extraction, and quality assessment of the included studies were performed by two independent reviewers. The results were reported as odds ratio (OR) with 95 % confidence intervals (CI).

**Results:**

A total of 52 studies with a combined 1,106,431 traumatic injury patients were included for quantitative analysis. The overall mortality rate was 6.77% (*n* = 74,930). When patients were treated in a non-trauma centre compared to a trauma centre, the pooled statistical odds of mortality were reduced (OR 0.74 [95% CI 0.69–0.79]; *p* < 0.001). When patients were treated in a non-trauma system compared to a trauma system the odds of mortality rates increased (OR 1.17 [95% CI 1.10–1.24]; *p* < 0.001). When patients were treated in a post-implementation/initial system compared to a mature system, odds of mortality were significantly higher (OR 1.46 [95% CI 1.37–1.55]; *p* < 0.001).

**Conclusion:**

The present study highlights that the survival of traumatic injured patients varies according to the stage of trauma system development in which the patient was treated. The analysis indicates a significant reduction in mortality following the introduction of the trauma system which is further enhanced as the system matures. These results provide evidence to support efforts to, firstly, implement trauma systems in countries currently without and, secondly, to enhance existing systems by investing in system development.

**Systematic review registration number:**

PROSPERO CRD42019142842.

**Supplementary Information:**

The online version contains supplementary material available at 10.1186/s13017-021-00381-0.

## Background

Injury remains the leading cause of death and disability [1, 2] and is responsible for 9% of mortality worldwide [3]. Injury also contributes to poor psychological, neuropsychological, and psychosocial instability [2, 4]. Due to the rate of death and disability arising from injuries, extensive efforts have been made to manage the response to trauma events. Initially, efforts to improve trauma care for the injured focused on prehospital training and triage, enhanced hospital training and procedures, and establishing in-hospital specialised trauma care teams [5–7]. These developments in trauma services then contributed to the implementation of trauma systems starting in the 1970s [8]. A trauma system provides care from prehospital care through to rehabilitation, as well as injury prevention, education, research, and quality programmes [9].

In recent decades, there has been a decrease in injury mortality rates as a result of comprehensive legislation pertaining to road safety and injury prevention campaigns [1]. Furthermore, the operationalisation and development of trauma systems has resulted in improved trauma care which has decreased patient morbidity and mortality [10–12]. Trauma systems are a cooperation across all health care providers to reduce preventable deaths and decrease morbidity and injury complications. This cooperation includes injury prevention programmes, coordination of care between prehospital and hospital contexts, rehabilitation care, and post-discharge care of trauma victims [13, 14].

A designated trauma centre/major trauma centre is a multi-specialty hospital that offers multiple levels of care for trauma patients. Trauma centres are often the first step in the implementation of a trauma system. While there is no agreed definition of a mature trauma system [15], it is widely accepted that it takes years for a system to mature and become an established part of the overall healthcare system once it has been implemented [16]. This paper has used the following three classifications to describe the stages of development of a trauma system: the establishment of a trauma centre, the establishment of a trauma system, and the maturation of the trauma system.

The different characteristics of trauma systems have been highlighted in prior reviews [16, 17]. Clinical outcomes, such as the mortality of injured patients at different stages of trauma system development, have not been examined previously. An understanding of the effectiveness of trauma systems at different stages can quantify the expected benefits yielded by trauma system implementation and development and is anticipated to inform investment in and continued enhancement of trauma systems. This study’s objective was to highlight the effectiveness of trauma systems in reducing victim mortality at the three stages of development: from trauma centres, to a formative system, and to a mature trauma system.

## Materials and methods

### Protocol and registration

This study was conducted in accordance with the Preferred Reporting Items for Systematic Reviews and Meta Analyses checklist for systematic reviews [18]. This review has been registered in the International Prospective Register of Systematic Reviews (PROSPERO) (registration number CRD42019142842). The protocol of this systematic review was published in BMJ Open [19].

### Eligibility criteria

#### Type of studies

Studies were included if they involved non-randomised control trials, observational studies, interrupted time series studies, controlled and non-controlled before and after studies, and prospective and retrospective cohort studies. Only peer-review population-based articles were included, and grey literature was excluded in this review. Articles for which full text was unavailable were excluded.

#### Participants/population

Traumatic injury individuals from all age groups, gender, and ethnicity with all causes of injury (such as road injury, falls, and cutting or piercing) were included. All levels of severity of traumatic injury patients, from relatively minor to severe, were included. Articles that dealt with ‘trauma/injury’ as a whole were included in this study as well as articles that focused on three or more injury types. Articles that focused on a specific type of injury such as whiplash or two or fewer specific types of injuries were excluded from this study and reflects that the majority of published trauma system evaluation studies represent all injury patients rather than focusing on a specific type of injury. Further, excluding studies that focused on a specific type of injury enhanced the ability of the review to generalise the study findings across all traumatic injury patients.

#### Interventions

Studies about the effectiveness of trauma care services in reducing mortality with sufficient data were included.

#### Comparators

Studies were eligible if they compared mortality rates for trauma patients treated at non-trauma centres and trauma centres, mortality rates between pre and post system implementation, as well as studies that evaluated system improvements made after the initial introduction of the trauma system. The timeframe included any period following initial establishment until the system was operating in a stable way. Noting that there is no agreed definition in the literature for system maturation [15], the operational definition of system maturation for the purpose of this review is any timepoint (without restriction) beyond the initial system formation.

#### Outcome measures

The primary outcome of this study was the mortality rate of injured patients treated in the three different stages of trauma system development.

### Information sources and search strategy

The search strategy was based on the recommended Joanna Briggs Institute (JBI) three-phase search progress [20]. An initial limited search was performed using the MEDLINE database followed by analysing the key words used in the title, abstract, and the index terms used to describe studies. A second search phase using all identified key words and index terms was implemented with the three included databases: MEDLINE, EMBASE, and CINAHL. Finally, additional articles were identified from other sources such as references of articles identified from database searching and author lists.

Included studies were limited to studies written in English, human-related studies, and published from January 1, 2000, to December 31, 2020. Since clinical healthcare and systems have evolved dramatically in the last three decades [21], and results for trauma centres and/ or systems before 2000 would add effects from out-of-date systems and clinical practice, the authors decided not to review the literature published before 2000. The primary author was contacted to obtain a full-text article when the full-text was not available. The keywords used in the search strategy of the selected databases to find relevant articles are shown in supplementary file, Appendix 1.

### Study selection

Citations identified through the search strategy were imported into Covidence systematic review software (Covidence, Melbourne, Australia). The Covidence software removed duplicate studies. Two independent reviewers (RA and SS) assessed the eligibility of every study by title/abstract screening and full-text screening. Any disagreement was resolved by a third reviewer. Articles that matched the study criteria were included in data extraction.

### Data collection process

Data extraction was performed by two independent authors (RA and SS). Disagreements were resolved through discussion and, if consensus could not be reached, by a third reviewer. Data extraction included author names, publication year, country of data origin, data collection period, source of data, design, sample size, characteristics of the study population, stage of trauma system development (centre; system; mature system) and years of operation, cause and type of trauma, level of injury severity, and mortality rate. No authors were contacted to clarify or obtain missing data or information.

### Quality and risk of bias assessments

The risk of bias of included studies was assessed using the Risk of Bias in Non-randomized Studies of Interventions (ROBINS-I) tool [22]. The tool considers bias due to confounding, selection of participants into the study, misclassification of interventions, deviations from intended interventions, missing data, measurement of outcomes, and selection of the reported result. Two independent reviewers assessed each study against rubrics provided by ROBINS-I. Disagreement was resolved first through discussion between the two reviewers, and then through consultation with the co-authors. Publication bias was visually assessed using a funnel plot. The quality of evidence was evaluated using the Grading of Recommendations, Assessment, Development and Evaluation (GRADE) criteria and a Summary of Findings was created using GRADEpro software (McMaster University, ON, Canada).

### Summary measures and synthesis of results

Included studies were pooled using statistical meta-analysis statistical software, Review Manager (RevMan) Version 5.4. (The Cochrane Collaboration, 2020)*.* For analysis calculation, inverse variance (IV) random effects models were used, effect sizes were expressed as odds ratios (OR) for dichotomous data, and their 95% confidence intervals (CI) for the data were explored. Given that the majority of studies in this field report an OR [16, 17, 23], an OR was utilised as the primarily outcome for the current study and is supplemented by the relative risk (RR) and absolute risk reduction (ARR) metrices. The choice of meta-analysis random model was considered according to Tufanaru et al. [24] and in consistent with previous systematic review [16]. The degrees of heterogeneity of intervention effects were considered using *I*^2^ values of 25%, 50%, and 75% correspond to low, moderate, and high, respectively [25].

The results were synthesised in three different groups (A, B, and C) according to the trauma system stage of development. A trauma centre is usually the first stage of system development. In group A of this review, studies were included that evaluated mortality rates for patients treated at non-trauma centres compared to trauma centres. Group B included studies reflecting the development of a network cooperating to establish a trauma system. In this group, studies that compared mortality rates between pre and post system implementation were included (second stage). In group C, mature trauma systems, studies were included that evaluated improvements after the initial introduction of the trauma system.

As factors that have been identified as predictors of mortality in a previous review [1], subgroups’ analyses were considered for variables such as age, gender, mechanism of injury, and level of injury severity, where there were sufficient studies providing data regarding the predictor and the level of heterogeneity was acceptable. A final subgroups synthesis was undertaken, including paediatric patients ≤ 18 years of old, road trauma patients, and severely injured patients with ISS ≥ 15.

## Results

### Study selection

**Fig. 1:**
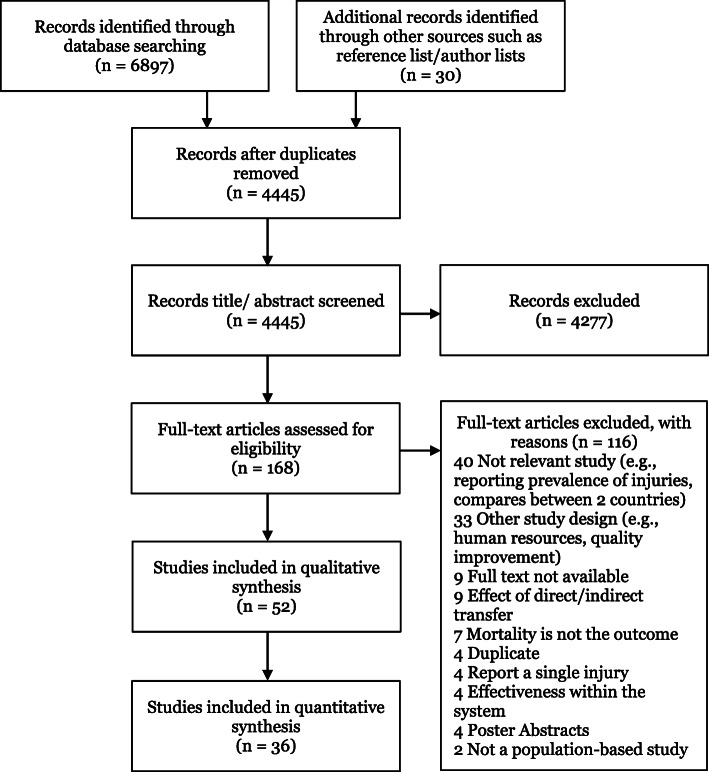
PRISMA flow diagram showing the searching and screening processes

A total of 6897 records were identified from the database search and 30 through reference list and author searching for a total of 6927 records. There were 4445 that were included after 2482 duplicates were removed. Following title and abstract review, a further 4277 records were excluded resulting in 168 papers eligible for full-text review. Of the 168 records, 52 articles met the study inclusion criteria and were included in the systematic review [26–77]. Of the 52, 36 studies were included in the quantitative analysis. The searching and screening processes are reported using a PRISMA flow diagram (Fig. 1).

### Study characteristics

The characteristics of included studies are shown in supplementary file, Appendix 2. In total, 27 (52%) of the included studies were published in the last 10 years (2011–2020). Of the included studies, 4 articles focused on paediatric patients (< 19 years) [36, 56, 66, 74], 3 articles on geriatric patients (≥ 65 years) [30, 44, 58], 1 article was focused on older adults (≥ 55 years) [70], and 13 articles included patients > 14 years [26, 28, 37, 41, 47, 48, 50, 53, 59, 67, 72, 75, 76], with the remaining studies (n = 31) inclusive of all age groups. Studies represented 12 different countries around the world with 28 (54%) from the USA and Canada. The remaining studies (n = 24) were conducted in Australia, China, Italy, Taiwan, Japan, Sweden, Korea, UK, Korea, and Israel. Five (9.6%) studies focused specifically on road trauma [37, 40, 42, 48, 58], 1 (1.9%) study was focused on road trauma and falls [35], and 42 (80.7%) studies had no restriction regarding the cause of the trauma. All articles were observational before-after and cohort studies utilising a retrospective and prospective method of data collection. Seventeen (32%) studies compared the mortality rate in the non-trauma centre and trauma centre group (group A) [29, 44, 52, 54–60, 62, 65, 66, 68–70, 76], 16 (30.7%) studies compared the mortality rate in a non-trauma system and post system group (group B) [26, 30, 33, 37, 41, 43, 45–48, 51, 63, 64, 67, 74, 77], and 19 (36.5%) studies compared the mortality rate in initial system implementation and mature systems (group C) [27, 28, 31, 32, 34–36, 38–40, 42, 49, 50, 53, 61, 71–73, 75].

### Risk of bias within studies

**Fig. 2:**
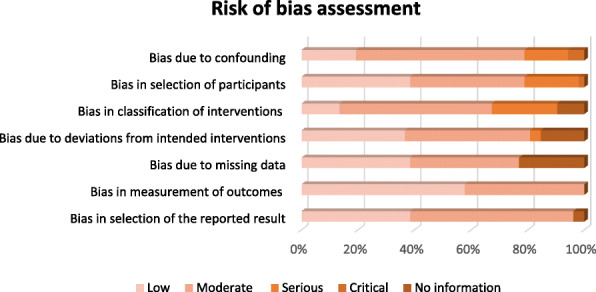
ROBINS-I overview risk of bias assessment

The risk of bias assessment is shown in Fig. 2. With respect to the confounding domains, 78% of the included studies were categorised as low to moderate risk. Participant selection bias was evident in some studies; 19% of studies were categorised as at serious risk of participant selection bias. Bias due to missing data was low to moderate in 77% of studies and not reported in 23% of the included studies. Bias in measurement of the outcomes was low or moderate in 58% and 42% of studies respectively. Bias in the reported result was low in 38% and moderate in more than half of the studies (57%). The overall risk of bias was moderate in 59% of these studies.

### Synthesis of results

A total of 52 studies, with combined 1,106,431 traumatic injury patients, were included in this systematic review. Of 1,106,431 patients, 6.77% died (*n* = 74,930) including patients in all 52 studies. Among patients in group A, 8.94% died in non-trauma trauma centres compared to 9.14% in trauma centres; an increase of 0.2% in the mortality rate was observed. When comparing mortality within group B, 6.56% died in non-trauma systems compared to 5.3% in trauma systems, while in group C, 8.59% died in early stage trauma systems compared to 5.36% in mature systems; a reduction by 1.26% and 3.23% of mortality rates was observed, respectively.

The meta-analysis included 36 studies: 10 studies for group A, 10 studies for group B, 14 studies for group C, and 2 studies for subgroup analysis (paediatric patients and patients with ISS > 15). The degree of heterogeneity among group A was moderate with *I*^2^ = 42% and low for groups B and C with *I*^2^ = 24% and 25%, respectively. The subgroup analyses presented with moderate to high heterogeneity: *I*^2^ = 55% for paediatric patients, *I*^2^ = 43% for road trauma patients, and *I*^2^ = 58% for patients with ISS > 15.

**Fig. 3:**
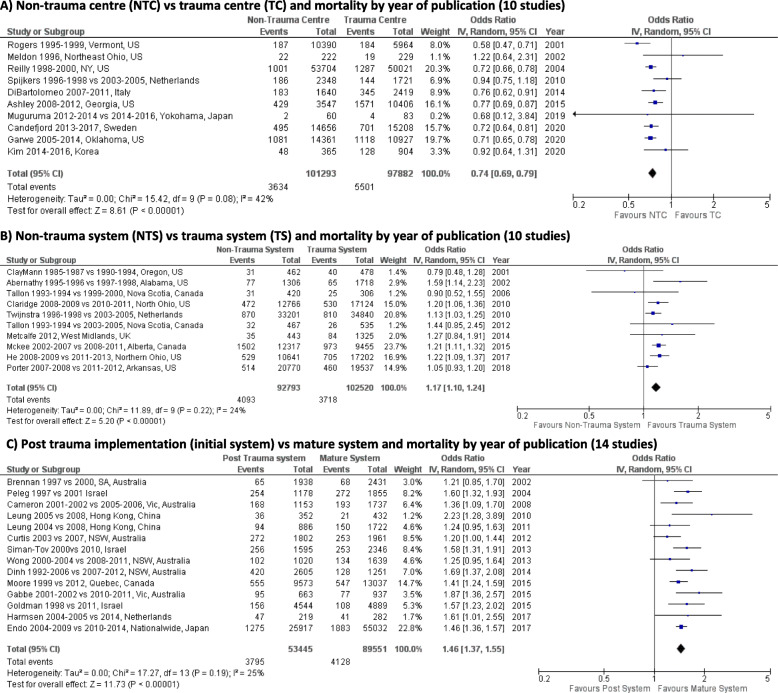
Meta-analyses of the association between the effectiveness of trauma systems at different stages of development and mortality. **A** Non-trauma centre (NTC) vs trauma centre (TC) and mortality by year of publication (10 studies). **B** Non-trauma system (NTS) vs trauma system (TS) and mortality by year of publication (10 studies). **C** Post trauma implementation (initial system) vs mature system and mortality by year of publication (14 studies)

The meta-analysis results for groups A, B, and C are shown in Fig. 3. When patients were treated in a non-trauma centre compared to a trauma centre (group A), pooled statistical odds of mortality were reduced (OR 0.74 [95% CI 0.69–0.79]; *p* < 0.001; RR = 0.76 or ARR = − 0.02). However, when patients were treated in a non-trauma system compared to a trauma system, the odds of mortality increased (group B), a difference that was statistically significant (OR 1.17 [95 % CI 1.10–1.24 p]; *p* < 0.001; RR = 1.16 or ARR = 0.01). Similarly, when patients were treated in an early stage system compared to a mature system (group C), odds of mortality increased with a difference that was statistically significant (OR 1.46 [95% CI 1.37–1.55]; *p* < 0.001; RR = 1.41 or ARR = 0.03).

**Fig. 4:**
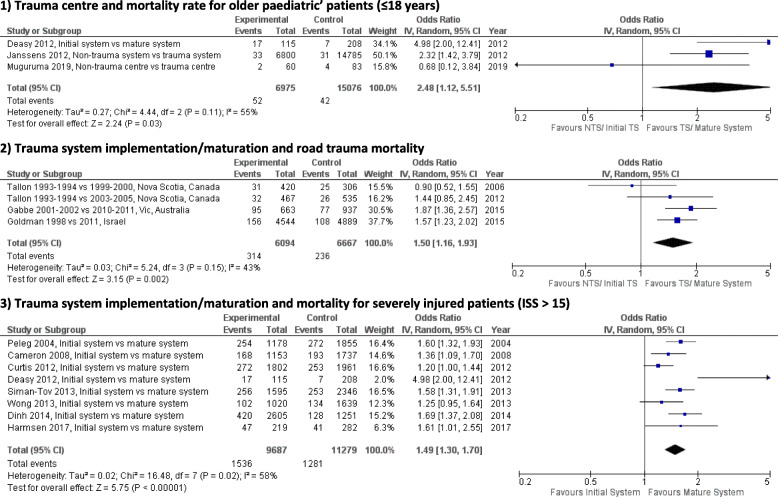
Mortality rates for different subgroup analysis at different stages of trauma system development. **1** Trauma centre and mortality rate for older paediatric’ patients (≤ 18 years). **2** Trauma system implementation/maturation and road trauma mortality. **3** Trauma system implementation/maturation and mortality for severely injured patients (ISS > 15)

The results of the subgroup analysis are shown in Fig. 4. When patients were treated in a non-trauma system or early stage system compared to a trauma system or mature system, the odds of mortality increased among paediatrics’ patients (≤ 18 years of old) (OR 2.48 [95% CI 1.12–5.51]; *p* < 0.05; RR = 2.42 or ARR = 0.03), among road trauma patients (OR 1.50 [95% CI 1.16–1.93]; *p* < 0.05; RR = 1.46 or ARR = 0.02), and among severely injured patients with ISS ≥ 15 (OR 1.49 [95% CI 1.30–1.70]; *p* < 0.001; RR = 1.41 or ARR = 0.05).

### Risk of bias across studies

**Fig. 5:**
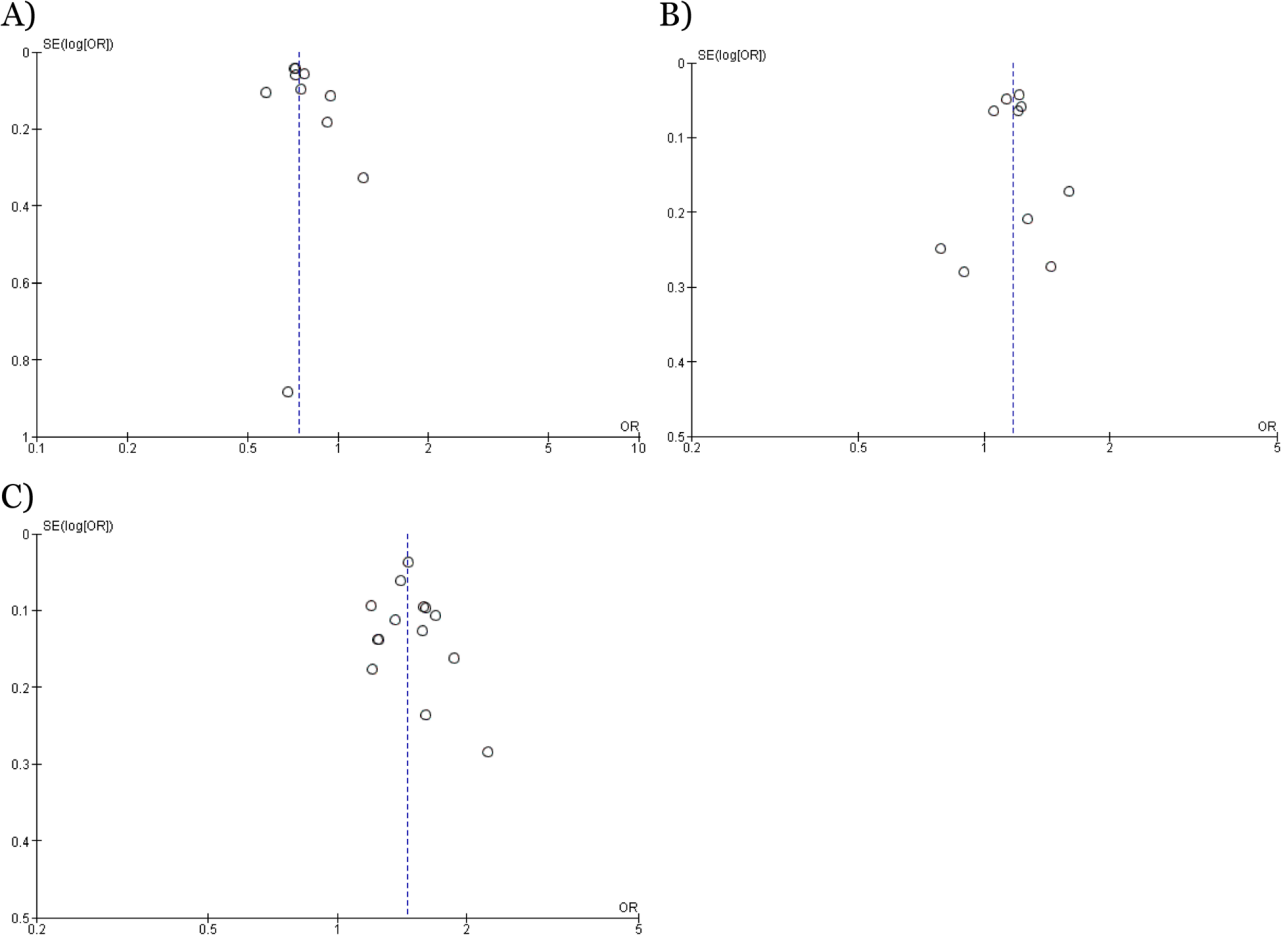
Funnel plot of meta-analysis assessing different stages of trauma system development and mortality rates. **A** Funnel plot for meta-analysis comparing mortality rate in the non-trauma centre vs trauma centre group. **B** Funnel plot for meta-analysis comparing mortality rate in the non-trauma system vs trauma system group. **C** Funnel plot for meta-analysis comparing mortality rate in initial system vs mature system

Publication bias was assessed visually with three funnel plots as shown in Fig. 5. The funnel plots showed relative symmetry indicating a moderate to low risk of publication bias in these meta-analyses. Therefore, the overall quality of evidence of this meta-analysis should be considered moderate. The GRADE certainty of evidence among the included studies was reported in supplementary file, Appendix 3. The quality of evidence was very low for trauma centre/trauma system and non-trauma system/non-trauma system studies (groups A and B), low for group C initial system and mature system studies, and moderate among road trauma patients treated at trauma centre/trauma system and non-trauma centre/non-trauma system subgroup analysis studies.

## Discussion

This systematic review of 52 studies involving over a million traumatic injury patients is to our knowledge the first meta-analysis examining the effect on mortality of trauma systems at different stages of development. The majority of included studies reported mortality rate as their primary outcome. For all patients in this study, the overall mortality rate was 6.77%. The mortality rate for patients treated in non-trauma centres or non-trauma systems was 7.7% compared to 7.1% for patients treated in a trauma centre or trauma system. With the inclusion of group C, early stage trauma system compared to mature systems, the mortality rate decreased from 7.83 to 6.73%. This pattern of progressive reduction in mortality rates highlights the importance of trauma system development and maturation.

In this analysis, the interpretation of the OR was frequently consistent with the other matrices used to analyse the data including the RR and ARR. There were no changes in the *p* value when we interpreted the data using OR, RR, or ARR among all group analyses. There was a significant reduction of absolute mortality risk of traumatic injury patients, particularly when considering the treatment of severely injured patients (ISS ≥ 15) in a mature system compared to care in an early stage system. This result reflects the effectiveness of prehospital triaging of such patients to a higher level of trauma care. The difference between pre event mortality rates across groups A, B, and C of trauma system development stages and post event mortality rate among paediatric patients was determined to be statistically significant, 0.75% and 0.28%, respectively. This suggests that trauma system development is as important for children as it is for adults. Road trauma mortality reduction was also observed following trauma system implementation and maturation. The degree of injury severity (ISS > 18) among road trauma patients was identified as a mortality predictive factor in a previous review [1].

The results of our study indicate that patient treatment at an institution that is part of a trauma system was observed to improve patients’ survival rates in the current study. Prior research has also determined trauma system care reduced prehospital time [77], reduced the length of hospital stay [28, 41], improved the overall health-related quality of life post-discharge [40], and had a lower mean cost of care [40, 45]. Furthermore, several studies have noted that there was an increase in traumatic injury patients accessing designated/major trauma centres following system implementation [39, 41] and more patients directly transported from the injury scene to a trauma centre [39, 40]. The increase in the number of patients accessing a trauma centre following the regionalisation of trauma systems has been linked to the effectiveness of prehospital triage by triaging the right patient to the right hospital [41, 78]. A previous systematic review found that transporting injured patients from the scene directly to an appropriate health care facility improved patient outcomes compared to patients who were transferred at a later time [79]. On the other hand, the increase in survival rate post-trauma system implementation could lead to a non-fatal burden as more injured patients would live with a long-term health-related issue such as physical and psychological problems. This reflects the necessity of continuing care following hospital discharge in order to achieve a rapid return to optimal health [80, 81]. The current study has offered further evidence as to the importance of continued development and maturation of trauma systems as more mature systems were associated with lower mortality compared to newly implemented systems.

Trauma service centralisation is usually the first phase in the development of a trauma system. This approach was seen in many high-income countries such as in North America [76], Europe [68], and Asia [69]. The majority of these countries have four to five different levels of trauma centres aligned with the American College of Surgeons Committee on Trauma (ASC-COT) criteria [9]. On the other hand, several studies have noted the absence of a developed trauma system in low- and middle-income countries (LMICs) [82, 83]. When studies examined the mortality rate between the different levels of trauma centres, evidence suggested that a lower mortality rate was observed among level I centres compared to level II [10, 84] and in level II compared to level III [76]. A previous systematic review determined that prehospital trauma systems in middle-income countries reduced traumatic injury mortality [85]. Reynolds et al. [86] also demonstrated a reduction of mortality when clinical protocols and trauma specialty care teams were available in LMICs. Further, a recent published study from the UAE showed that trauma system developments such as establishing a Trauma Committee and Registry to enhance strategies for injury prevention contributed to reducing traumatic injury by 56% over a decade [87]. Such findings highlight and support the potential success of trauma system development in LMICs.

Although the current study did not find a reduction in mortality rates between trauma centres and non-trauma centres, logistically, this is a first and important step toward organising a regional trauma system. This is particularly important for LMICs, where limited resources can be a barrier to establishing a state-wide/national wide trauma system. While the difference was determined to be statistically significant, the clinical significance of this difference is relatively small, as patients with more severe injuries are likely to be treated at trauma centres than non-trauma centres. Our results provide evidence to justify and prompt healthcare providers and trauma system policymakers’ commitment to continual refinement of trauma systems and shape ongoing government investment in system development. Understanding the effectiveness of trauma systems at different stages of development provides support for LMICs, where system development is still emerging.

This meta-analysis has several limitations. First, our search strategy was limited to peer review published articles and excluded grey literature; thus, some healthcare providers and government reports would have been excluded. Second, some studies reported adjusted risk mortality while others reported unadjusted risk and the variation could lead to statistical differences in our meta-analysis. Third, our study found that when patients were treated in a non-trauma centre compared to a trauma centre, the odds of mortality were reduced. However, these statistical differences could be primarily related to selection bias as patients with more severe injuries were more likely to be treated at trauma centres than non-trauma centres. Finally, some subgroup’s analyses were not possible due to the high level of heterogeneity or absence of data pertaining to these predictor variables. Among the included studies, moderate heterogeneity was found for group A; however, groups B and C presented with low degree of heterogeneity.

## Conclusion

This comprehensive literature review and meta-analysis summarises the findings of trauma system improvement over the last two decades. Management of traumatically injured patients, particularly patients with high injury severity, in a mature trauma system was associated with decreased mortality compared with management of these patients in a non-trauma system. The results of this review provide evidence to support efforts to implement such a system in LMICs and encourage countries with existing systems to further invest in system development.

## Supplementary Information

**Additional file 1: Supplemental file, Appendix 1.**

**Additional file 2: Supplemental file, Appendix 2.**

**Additional file 3: Supplemental file, Appendix 3.**

## Data Availability

Not applicable.

## Abbreviations

PRISMA: Preferred Reporting Items for Systematic Reviews and Meta Analyses
JBI: Joanna Briggs Institute
ROBINS-I: Risk of Bias in Non-randomized Studies of Interventions
OR: Odds ratios
ARR: Absolute risk reduction
RR: Relative risk
CI: Confidence intervals
ISS: Injury severity score
LMIC: Low- and middle-income countries
GRADE: Grading of Recommendations, Assessment, Development and Evaluation
