# Supplementary material for: The effectiveness of trauma care systems at different stages of development in reducing mortality: a systematic review and meta-analysis
Source: World J Emerg Surg. 2021 Jul 13;16:38. doi: 10.1186/s13017-021-00381-0 (PMC8278750; doi:10.1186/s13017-021-00381-0)
Supplement: Supplementary file 1 — Additional file 1: Supplemental file, Appendix 1. [file 13017_2021_381_MOESM1_ESM.docx]

**Supplemental file, Appendix 1:** Search strategy for MEDLINE

| Search strategy for MEDLINE (Ovid) |
| --- |
| #1 (trauma* or 'major trauma*' or injur* or 'major injur*' or 'major disabilit*' or 'trauma patient*' or 'injury patient*' or 'injured patient*' or 'traumatic injury*' or 'multiple trauma' or 'multiple traumatic injur' or 'injury prevention').mp. [mp=title, abstract, original title, name of substance word, subject heading word, floating sub-heading word, keyword heading word, organism supplementary concept word, protocol supplementary concept word, rare disease supplementary concept word, unique identifier, synonyms] |
| #2 ('trauma system*' or 'major trauma center*' or 'non-trauma center*' or ‘or 'trauma centre*' or 'trauma registry*' or 'trauma care*' or 'trauma service*' or 'metro trauma service*' or 'regional trauma service*' or 'rural trauma service*' or 'trauma prevention*' or 'services for trauma' or 'trauma evaluation*' or 'evolution of trauma' or 'implementation of trauma*' or 'trauma implementation*' or 'trauma introduction*' or 'introduction of trauma*').mp. [mp=title, abstract, original title, name of substance word, subject heading word, floating sub-heading word, keyword heading word, organism supplementary concept word, protocol supplementary concept word, rare disease supplementary concept word, unique identifier, synonyms] |
| #3 ('death*' or 'mortality*' or 'fatal mortality' or 'hospital mortality' or 'survival*' or 'quality of life*' or 'functional outcome*' or 'length of hospital stay' or 'recovery' or 'population wellbeing' or 'health outcome*' or 'patient recovery*' or 'patient outcome*' or 'recovery period').mp. [mp=title, abstract, original title, name of substance word, subject heading word, floating sub-heading word, keyword heading word, organism supplementary concept word, protocol supplementary concept word, rare disease supplementary concept word, unique identifier, synonyms] |
| #4 1 and 2 and 3 |
| #5 limit 4 to (english language and humans and yr="2000 - 2020") |
