# Supplementary material for: The effectiveness of trauma care systems at different stages of development in reducing mortality: a systematic review and meta-analysis
Source: World J Emerg Surg. 2021 Jul 13;16:38. doi: 10.1186/s13017-021-00381-0 (PMC8278750; doi:10.1186/s13017-021-00381-0)
Supplement: Supplementary file 2 — Additional file 2: Supplemental file, Appendix 2. [file 13017_2021_381_MOESM2_ESM.docx]

**Supplemental file, Appendix 2:** Characteristics of included studies

| **Author & year** | **Country of data**  **origin** | **Stage of trauma**  **System development** | **System years**  **of operation** | **Data collection**  **Period** | **Method** | **Age group** | **Cause of trauma** | **Level of Injury severity** | **Outcome (mortality %)** |
| --- | --- | --- | --- | --- | --- | --- | --- | --- | --- |
| Group A - Studies compared between non-trauma centres and trauma centres | | | | | | | | | |
| DiRusso 2001 | New York, USA | NTC vs TC | 1999 | 1994 vs 1998 | Before and after study | All age group | All cause of trauma | - | Pre: 8.20  Post: 6.10 |
| Rogers 2001 | Vermont, USA | NTC vs TC | - | 1995 - 1999 | Observational | All age group | All cause of trauma | ICD-9-CM code of 800–959.9 | Pre: 1.80  Post: 3.10 |
| Meldon 2002 | Northeast Ohio, USA | NTC vs TC | - | 1996 | Observational | > 80 years | All cause of trauma | ICD-9-CM code of 800–959.9 | Pre: 9.91  Post: 8.30 |
| Reilly 2004 | New York, USA | NTC vs TC | - | 1998 – 2000 | Observational | All age group | All cause of trauma | ICD-9-CM code of 800–959.9 | Pre: 1.86  Post: 2.57 |
| Scheetz 2005 |  | NTC vs TC | - | 2000 | Observational | 65 - 99 years | Road trauma | ISS ≥ 16 | Pre: 2.62  Post: 11.51 |
| Demetriades 2006 | USA | NTC vs TC | - | 1994 - 2008 | Observational | > 14 years | All cause of trauma | ISS > 15 | Pre: 18.21  Post: 15.00 |
| Pracht 2008 | Florida, USA | NTC vs TC | 1980s | 1995 – 2004 | Observational | 0 - 19 years | All cause of trauma | - | Pre: 1.71  Post:4.23 |
| Arthur 2009 | 7 states, USA | NTC vs TC | - |  | Cohort | < 80 years | All cause of trauma | - | Pre: 10.69  Post: 7.93 |
| Haas 2009 | USA | NTC vs TC | - | 2001 - 2002 | Cohort | < 65 years | All cause of trauma | ISS > 15 | Pre: 28.40  Post: 22.60 |
| Spijkers 2010 | Utrecht, Netherlands | NTC vs TC | 1999 | 1996 - 1998 vs 2003 - 2005 | Before and after study | ≥ 18 years | All cause of trauma | All level of injury severity | Pre: 7.92  Post: 8.37 |
| DiBartolomeo 2014 | Italy | NTC vs TC | 2006 | 2007 – 2011 | Observational | > 1 years | All cause of trauma | ISS > 15 | Pre: 11.16  Post: 14.26 |
| Harnod 2014 | Nationalwide, Taiwn | NTC vs TC | - | 2006 – 2008 | Observational | All age group | All cause of trauma | ISS ≥ 15 | Pre: 13.41  Post: 11.05 |
| Ashley 2015 | Georgia, USA | NTC vs TC  NTC vs TC | - | 2008 – 2012 | Observational | All age group | All cause of trauma | ICISS of < 0.85 | Pre: 12.10  Post: 15.10 |
| Muguruma 2019 | Yokohama, Japan | NTC vs TC | - | 2012 - 2014 vs 2014-2016 | Observational | < 16 years | All cause of trauma | ISS ≥ 16 | Pre: 3.33  Post: 4.82 |
| Kim 2020 | Korea | NTC vs TC | - | 2014 – 2016 | Before and after study | All age group | All cause of trauma | ISS ≥ 15 | Pre: 13.15  Post: 14.16 |
| Garwe 2020 | Oklahoma, USA | NTC vs TC | - | 2005 – 2014 | Cohort | ≥ 55 years | All cause of trauma | ISS ≥ 16 | Pre: 7.53  Post: 10.23 |
| Candefjord 2020 | Sweden | NTC vs TC | - | 2013 – 2017 | Cohort | All age group | All cause of trauma | NISS > 15 | Pre: 3.38  Post: 4.61 |
| Group B – Studies compared between non-trauma system and trauma system | | | | | | | | | |
| Claridge 2013 | North Ohio, USA | NTS vs TS | 2010 | 2008 – 2009 vs 2010 – 2011 | Before and after study | All age group | All cause of trauma | ISS > 14 | Pre: 3.70  Post: 3.10 |
| Abernathy 2002 | Alabama, USA | NTS vs TS | 1996 | 1995 - 1996 vs 1997 – 1998 | Before and after study | All age  Group | All cause of trauma | ICD-9-CM code of 800–959.9 | Pre: 5.90  Post: 3.78 |
| ClayMann 2001 | Oregon, USA | NTS vs TS | - | 1985 - 1987 vs 1990 – 1994 | Cohort | < 80 years | All cause of trauma | ICD-9-CM code of 800–959.9 | Pre: 6.71  Post: 8.37 |
| He 2017 | Northern Ohio, USA | NTS vs TS | 2010 | 2008 - 2009 vs 2011 – 2013 | Cohort | All age group | All cause of trauma | - | Pre: 4.97  Post: 4.10 |
| He 2016 | Northern Ohio, USA | NTS vs TS | 2010 | 2006 – 2012 | Cohort | ≥ 15 years | All cause of trauma | - | Pre: 5.30  Post: 5.03 |
| Janssens 2012 | Netherlands | NTS vs TS, regionalisation model | 1999/2000 | 1996 - 1998 vs 2001 - 2006 | Before and after study | ≤ 18 years | All cause of trauma | - | Pre: 0.49  Post: 0.21 |
| Mann 2001 | Washington, USA | NTS vs TS | 1993 | 1988 - 1992 vs 1993 – 1995 | Before and after study | > 65 years | All cause of trauma | Any injury ICD-9-CM code of 800–959.9 | Pre: 10.10  Post: 10.40 |
| Mckee 2015 | Alberta, Canada | NTS vs TS, all-inclusive model | 2008 | 2002 - 2007 vs 2008 - 2011 | Before and after study | ≥ 16 years | All cause of trauma | ISS ≥ 12 | Pre: 12.20  Post: 10.30 |
| Metcalfe 2014 | West Midlands, UK | NTS vs TS, regional trauma network model | - | 2012 (within first 6-month) | Observational | All age group | All cause of trauma | - | Pre: 7.90  Post: 6.34 |
| Metcalfe 2016 | England, UK | NTS vs TS, regionalisation model | 2012 | 2012 (270 days before vs 270 days after) | Before and after study | All age group | All cause of trauma | - | Pre: 8.38  Post: 6.45 |
| Porter 2018 | Arkansas, USA | NTS vs TS | 2009 | 2007 - 2008 vs 2011 - 2012 | Before and after study | > 15 & older | All cause of trauma | - | Pre: 2.47  Post: 2.35 |
| Tallon 2006 | Nova Scotia, Canada | NTS vs TS, 2-year after | 1998 | 1993 - 1994 vs 1999 – 2000 | Before and after study | ≥ 16 years | Road trauma | - | Pre: 7.38  Post: 8.17 |
| Tallon 2012 | Nova Scotia, Canada | NTS vs TS, 10-year after - mature system | 1998 | 1993 - 1994 vs 2003 – 2005 | Observational | ≥ 16 years | Road trauma | - | Pre: 6.85  Post: 4.86 |
| Tinkoff 2010 | Delaware, USA | NTS vs TS, all -inclusive model | 2000 | 1998 - 1999 vs 2006 – 2007 | Before and after study | All age group | All cause of trauma | - | Pre: 4.18  Post: 1.23 |
| Twijnstra 2010 | Utrecht, Netherlands | NTS vs TS, all -inclusive model | 1999 | 1996 - 1998 vs 2003 – 2005 | Before and after study | All age group | All cause of trauma | - | Pre: 2.62  Post: 2.32 |
| Utter 2006 | 24 states, USA | Exclusive Systems vs most inclusive systems | - | 2001 | Cohort | > 15 years | All cause of trauma | ISS ≥ 16 | Pre: 14.60  Post: 14.70 |
| Group C - Studies evaluated improvements subsequently to the initial introduction of the trauma system | | | | | | | | | |
| Curtis 2012 | NSW, Australia | TS - Post system introduction, further improvement | 1992 | 2003 vs 2007 | Cohort | ≥ 15 years | All cause of trauma | ISS ≥ 15 | Initial: 15.09  Mature: 12.90 |
| Wong 2013 | NSW, Australia | TS - Post system introduction, further improvement | 1992 | 2000 - 2004 vs 2008 - 2011) | Cohort | ≥ 15 years | All cause of trauma | ISS ≥ 15 | Initial: 10.00  Mature: 8.18 |
| Leung 2010 | Hong Kong, China | TS - Post intro study - 1 centre | 2003 | 2005 vs 2008 | Observational | All age group | All cause of trauma | - | Initial: 10.50  Mature: 4.90 |
| Leung 2011 | Hong Kong, China | TS - Post system introduction, 5 centres | 2003 | 2004 vs 2008 | Observational | All age group | Road trauma & fall | - | Initial: 10.61  Mature: 8.71 |
| Liberman 2004 | Quebec, Canada | TS - Post system introduction, first year vs 13 years post intro | 1993 | 1992 - 1993 vs 2001 - 2002 | Observational | All age group | All cause of trauma | ISS > 12 | Initial: 51.8  Mature: 8.60 |
| Moore 2015 | Quebec, Canada | TS - Post system introduction, all-inclusive model – 12 years | 1996 | 1999 vs 2012 | Cohort | ≥ 16 years | All cause of trauma | - | Initial: 5.80  Mature: 4.20 |
| Harmsen 2017 | Netherlands | TS - Post system introduction | - | 2004 - 2005 vs 2015 | Cohort | All age group | All cause of trauma | ISS ≥ 15 | Initial: 21.46  Mature: 14.54 |
| Moore 2017 | Nationalwide, Canada | TS - Post system introduction, all-inclusive model | 1990s | 2006 vs 2012 | Cohort | ≥ 16 years | All cause of trauma | ISS >12 | Initial: 11.01  Mature: 10.76 |
| Peleg 2004 | Israel | TS - Post system introduction | - | 1997 vs 2001 | Cohort | All age group | All cause of trauma | ISS >16 | Initial: 21.60  Mature: 14.70 |
| Barquist 2000 | Finger Lakes New York, USA | TS - Post system introduction | 1993 | 1993 - 1994 vs 1995 – 1996 | Observational | All age group | All cause of trauma | ISS ≥ 9 | Initial: 8.27  Mature: 7.21 |
| Brennan 2002 | SA, Australia | TS - Post system introduction | 1997 | 1997 vs 2000 | Observational | All age group | All cause of trauma | ICD-9-CM code of 800–959.9 | Initial: 3.40  Mature: 2.80 |
| Cameron 2008 | Victoria, Australia | TS - Post system introduction | 2000/2001 | 2001 - 2002 vs 2005 - 2006 | Cohort | All age group | All cause of trauma | ISS > 15 | Initial: 14.57  Mature: 11.11 |
| Deasy 2012 | Victoria, Australia | TS - Post system introduction, all-inclusive model – 10 years | 2000/2001 | 2002 vs 2009 | Cohort | < 18 years | All cause of trauma | ISS > 15 | Initial: 14.78  Mature: 3.37 |
| Dinh 2014 | NSW, Australia | TS - Post system introduction further improvement | 1992 | 1992 - 2006 vs 2007 – 2012 | Cohort | ≥ 15 years | All cause of trauma | ISS > 15 | Initial: 16.12  Mature: 10.23 |
| Dutton 2010 | Maryland, USA | TS - Post system introduction, further improvment | 1990s | 1997 vs 2008 | Observational | All age group | All cause of trauma | - | Initial: 3.00  Mature: 3.70 |
| Siman-Tov 2013 | Israel | TS - Post system introduction | - | 2000 vs 2010 | Cohort | All age group | All cause of trauma | ISS ≥16 | Initial: 16.10  Mature: 10.80 |
| Endo 2017 | Nationalwide, Japan | TS - Post system introduction | 2003 | 2004 - 2009 vs 2010 - 2014 | Observational | All age group | All cause of trauma | AIS ≥ 3 | Initial: 4.92  Mature: 3.42 |
| Gabbe 2015 | Victoria, Australia | TS - Post system introduction study, all-inclusive model | 2001 | 2001 -2002 vs 2010 – 2011 | Cohort | All age group | Road trauma | ISS >12 | Initial: 14.33  Mature: 8.22 |
| Goldman 2015 | Israel | TS - Post system introduction | 1990s | 1998 vs 2011 | Cohort | All age group | Road trauma | ICD-9-CM code of 800–959.9 | Initial: 3.43  Mature: 2.21 |

***Note:*** TC: Trauma Centre; TS: Trauma System; MTS: Mature Trauma System; NTC: Non-Trauma Centre; NTS: Non-Trauma System; ITS: Initial Trauma System; CI: Confidence interval; OR: Odds ratio; ISS: injury severity score; ICD-9-CM: International Classification of Diseases, 9th Revision, Clinical Modification
