# Supplementary material for: The effectiveness of trauma care systems at different stages of development in reducing mortality: a systematic review and meta-analysis
Source: World J Emerg Surg. 2021 Jul 13;16:38. doi: 10.1186/s13017-021-00381-0 (PMC8278750; doi:10.1186/s13017-021-00381-0)
Supplement: Supplementary file 3 — Additional file 3: Supplemental file, Appendix 3. [file 13017_2021_381_MOESM3_ESM.docx]

**Supplemental file, Appendix 3:** GRADE quality of evidence among the group analysis

| **Outcome** | **Anticipated absolute effects (95% CI)** | | **Relative effect (95% CI)** | **№ of participants  (studies)** | **Certainty of the evidence (GRADE)** |
| --- | --- | --- | --- | --- | --- |
|  | **Risk with TC/TS/MTS** | **Risk with NTC/NTS/ITS** |  |  |  |
| Mortality among patients treated at TC and NTC | 56 per 1,000 | 42 per 1,000 (39 to 45) | OR 0.74 (0.69 to 0.79) | 199175 (10 observational, cohort, before-after-studies) | ⨁◯◯◯  VERY LOW |
| Mortality among patients treated at TS and NTS | 36 per 1,000 | 42 per 1,000 (40 to 45) | OR 1.17 (1.10 to 1.24) | 195313 (10 observational, cohort, before-after-studies) | ⨁◯◯◯  VERY LOW |
| Mortality among patients treated at MTS and ITS | 46 per 1,000 | 66 per 1,000 (62 to 70) | OR 1.46 (1.37 to 1.55) | 142996 (14 observational, cohort studies) | ⨁⨁◯◯  LOW |
| Mortality among younger patients treated at TC and NTC | 3 per 1,000 | 7 per 1,000 (3 to 15) | OR 2.48 (1.12 to 5.51) | 22051 (3 observational) | ⨁⨁◯◯  LOW |
| Mortality among road trauma patients treated at TC/TS and NTC/NTS | 35 per 1,000 | 52 per 1,000 (41 to 66) | OR 1.50 (1.16 to 1.93) | 12761 (4 observational, before-after-studies) | ⨁⨁⨁◯  MODERATE |
| Mortality among patients with ISS ≥15 treated at MTS and ITS | 114 per 1,000 | 160 per 1,000 (143 to 179) | OR 1.49 (1.30 to 1.70) | 20966 (8 observational studies) | ⨁◯◯◯  VERY LOW |
| ***Note:*** TC: Trauma Centre; TS: Trauma System; MTS: Mature Trauma System; NTC: Non-Trauma Centre; NTS: Non-Trauma System; ITS: Initial Trauma System; CI: Confidence interval; OR: Odds ratio; ISS: injury severity score. | | | | | |

**Certainty of the evidence (GRADE) Grade Definition**

**High:** Further research is very unlikely to change our confidence in the estimate of effect.

**Moderate:** Further research is likely to have an important impact on our confidence in the estimate of effect and may change the estimate.

**Low:** Further research is very likely to have an important impact on our confidence in the estimate of effect and is likely to change the estimate.

**Very low:** Any estimate of effect is very uncertain.
